# Updating mortality risk estimation in intensive care units from high-dimensional electronic health records with incomplete data

**DOI:** 10.1186/s12911-023-02264-7

**Published:** 2023-08-30

**Authors:** Bertrand Bouvarel, Fabrice Carrat, Nathanael Lapidus

**Affiliations:** 1 Sorbonne Université, INSERM, Institut Pierre Louis d’Epidémiologie et de Santé Publique IPLESP, Paris, F75012 France; 2 AP-HP.Sorbonne Université, Public Health Department, Saint-Antoine Hospital, Paris, F75012 France

**Keywords:** Clinical decision support systems, Electronic health records, Machine learning, Multiple imputation, Neural network

## Abstract

**Background:**

The risk of mortality in intensive care units (ICUs) is currently addressed by the implementation of scores using admission data. Their performances are satisfactory when complications occur early after admission; however, they may become irrelevant in the case of long hospital stays. In this study, we developed predictive models of short-term mortality in the ICU from longitudinal data.

**Methods:**

Using data collected throughout patients’ stays of at least 48 h from the MIMIC-III database, several statistical learning approaches were compared, including deep neural networks and penalized regression. Missing data were handled using complete-case analysis or multiple imputation.

**Results:**

Complete-case analyses from 19 predictors showed good discrimination (AUC > 0.77 for several approaches) to predict death between 12 and 24 h onward, yet excluded 75% of patients from the initial target cohort, as data was missing for some of the predictors. Multiple imputation allowed us to include 70 predictors and keep 95% of patients, with similar performances.

**Conclusion:**

This proof-of-concept study supports that automated analysis of electronic health records can be of great interest throughout patients’ stays as a surveillance tool. Although this framework relies on a large set of predictors, it is robust to data imputation and may be effective early after admission, when data are still scarce.

**Supplementary Information:**

The online version contains supplementary material available at 10.1186/s12911-023-02264-7.

## Introduction

Intensive care units (ICUs) admit critically ill patients who require constant care and supervision from life support equipment and medication to ensure normal bodily functions [1]. The illness severity of patients explains the high fatality rate in ICUs, which remains at approximately 20% globally [2]. Another explanation for this very high mortality rate lies in the rapid evolution of patients’ conditions and the risk of delayed management of complications. Thus, timely diagnosis and relevant management and treatment are crucial to amend prognosis. To address this issue and identify patients with the highest risks of severe complications, prognostic scores have been developed, such as the Acute Physiology And Chronic Health Evaluation II (APACHE II) [3], the Simplified Acute Physiology Score (SAPS II) [4] or the Sequential Organ Failure Assessment Score (SOFA) [5], used to predict in-hospital mortality from data collected upon admission or in the first 24 h in the ICU. These prediction scores, however, have a number of limitations, one of the most important being that they rely on patients’ data at admission, without re-evaluation during their stays, as for most published ICU mortality prediction methods [6]. The prediction performances of these scores are therefore high regarding early complications but show a decrease in their capacity to estimate the mortality risk in patients who have already spent several days or weeks in the ICU [7].

To address this issue, other scoring systems have been developed to estimate the risk of complications throughout the stay using updated collection of patient data. Regarding the risk of septic shock, one of the leading causes of death in ICU patients, longitudinal collection of data thus allowed to identify a “pre-shock” state during which the symptoms of the upcoming failure are not yet clinically visible [8]. Early management of this state may prevent the occurrence of septic shock and improve survival. Opportunities to predict or identify the onset of complications early therefore represent a major challenge in the management of ICU patients. The current spread of health care data warehouses offers new opportunities to closely monitor the evolution of ICU inpatients and to develop prognostic scores relying on a wider range of data [9, 10]. These databases enable the collection and centralization of detailed data throughout inpatients’ stays via demographic characteristics, physiological measures, diagnoses, laboratory analyses, medical imaging, medical notes, etc. ICUs are highly monitored environments and important data sources for these warehouses. Repeated collection of data allows us to study the evolution of patient characteristics and to identify factors associated with the occurrence of worsening conditions, possibly leading to complications or death. Appropriate machine learning algorithms are required to address the massive amount of data available in these warehouses. Deep learning methods have been extensively studied in recent years for their abilities to manage large amounts of data, and specific architectures of deep learning networks, such as convolutional and recurrent neural networks, have been developed to handle longitudinal data [9, 11]. Such predictive modeling approaches may, however, present limited interest when their use relies on a large number of predictors, several of which may be unavailable in some patients.

Considering recent advances in predictive modeling from longitudinal data using neural networks [12, 13], we aimed to develop and validate models predicting ICU mortality for higher lengths of stay than those well evaluated by the existing scores. These models were built from ICU hospitalizations lasting more than 48 h, using longitudinal health care data with missing values from electronic health records available in the freely accessible Medical Information Mart for Intensive Care (MIMIC-III) critical care database [14, 15]. Different architectures of deep learning neural networks were evaluated in the context of missing values for some predictors and compared with predictive models based on penalized regression.

## Materials and methods

### Data collection and preparation

All predictive models were trained from the MIMIC-III database (version: January 2020). This data warehouse is an open-access database that collected anonymized care data in 46,520 patients from 19 critical care units of the Beth Israel Deaconess Medical Center in Boston, USA, between 2001 and 2012. Only the first ICU stay of each patient in the MIMIC-III database was used. Patients aged over 100 years were excluded, as well as patients under 15 in order not to mix pediatric patients with adult patients and to keep a homogeneous population, as their conditions, risk factors and vital prognosis highly differ. Patients with missing information on vital status at hospital discharge and those with an ICU length of stay lower than 48 h were also excluded, as the method requires the collection of data over 36 h to predict mortality after a 12-hour gap following the end of this observation period.

**Fig. 1:**
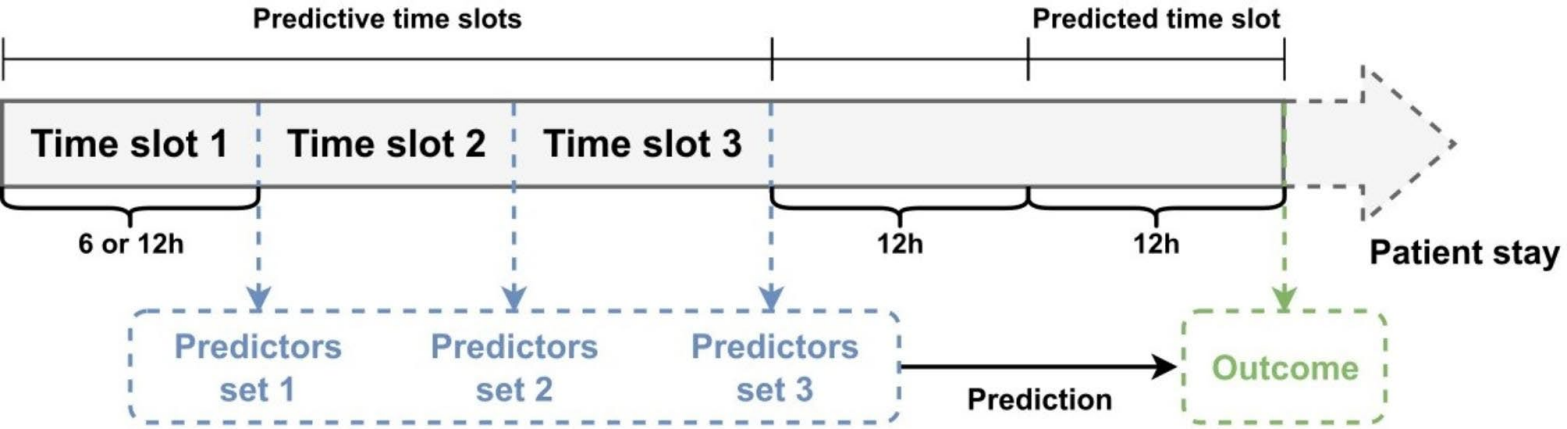
Time-slot formatting of data. For variables with values evolving within a predictive time slot, the latest values were used. Durations of 6 and 12 h were compared for predictive time slots

Data collected throughout patients’ stays were split into several time slots, during which information was summarized by a unique value per variable (the latest value collected during each time slot) (Fig. 1). Short-term evolution of all patients’ characteristics was accounted for with the use of triplets for consecutive values over three time slots; these triplets were used as predictors for model development. Formats of 6- and 12-hour time slots were compared, with predictions still addressing mortality between 12 and 24 h following the 3rd predictive slot to find a trade-off between the ability to capture short-term evolutions and the overall duration of data collection. An additional format with 6 consecutive 6-hour predictive time slots was also tested. In all analyses, the models aimed at predicting mortality after a 12-hour gap following the third predictive time slot. For instance, using 12-hour time slots following time t_0_, information collected over the 3 time slots between t_0_ and t_0_ + 36 h was used to predict mortality between t_0_ + 48 h and t_0_ + 60 h. The 12-hour gap between t_0_ + 36 h and t_0_ + 48 h was considered clinically relevant, as it is short enough to predict upcoming lethal complications yet leaves some time for physicians to become aware of possibly undetected complications and modify diagnostic or therapeutic management if necessary. Unlike the current scores using admission data, these models therefore apply only to patients staying more than 48 h in the ICU.

The predicted endpoint was mortality in the ICU, coded as a binary variable. Assuming that patients’ characteristics associated with mortality in the ICU were mostly identified by previously published prognostic scores, we first developed models relying only on variables used in the APACHE II and SAPS II scores [3, 4] to predict mortality, as well as the SOFA score [5] to predict the occurrence of organ failure. Nineteen predictors used in these scores were selected (Table 1), including medical history, vital signs, and blood tests, as well as administrative features such as previous hospitalization wards, which can provide information on the most common complications.

To assess the relevance of using longitudinal data, predictive models derived solely from admission data were built from the same dataset (thus only addressing patients alive and still in the ICU 48 h after admission to predict death anytime during their stays).

**Table 1:** Characteristics of patients in the Complete-case and Imputed-19 cohorts at admission (median [Q1, Q3])

			Complete-case				Imputed-19	
		Missing data	Survivors n = 3489	Non-survivors n = 787		Missing data	Survivors n = 8590	Non-survivors n = 1499
Admission type		0%	- ELEC: 16.4% - EMER: 81.4% - URG: 2.2%	- ELEC: 5.1% - EMER: 92.4% - URG: 2.6%		0%	- ELEC: 15.0% - EMER: 80.7% - URG: 4.3%	- ELEC: 4.7% - EMER: 90.0% - URG: 5.3%
Previous ward		0%	17 classes	17 classes		0%	17 classes	17 classes
Current ward		0%	18 classes	18 classes		0%	18 classes	18 classes
Age	years	0%	64.66 [51.98, 75.94]	69.99 [56.43, 80.07]		0%	65.2 [52.05, 76.56]	70.98 [57.69, 80.32]
PaO_2_	mmHg	0%	133 [96, 202]	129 [92, 198.5]		15.65%	163 [98, 285]	128 [83, 209]
FiO_2_	proportion	0%	0.5 [0.4, 0.7]	0.5 [0.5, 0.8]		22.07%	0.6 [0.5, 1]	0.7 [0.5, 1]
GCS	points	0%	9 [6, 15]	7 [4, 11]		0.65%	11 [6, 8]	10 [6, 8]
MAP	mmHg	0%	79 [70, 90]	77 [67, 90]		21.45%	80 [70, 92]	78 [68, 91.75]
Platelets	10^3^ cells/mm^3^	0%	188 [135, 260]	190 [115, 266.5]		0.06%	198 [141, 267]	190 [115, 269.75]
Blood creatinine	mg/dL	0%	0.9 [0.7, 1.4]	1.1 [0.8, 1.8]		0.04%	0.9 [0.7, 1.3]	1.1 [0.8, 1.9]
Heart rate	min^− 1^	0%	86 [75, 99]	88 [74.5, 103]		0.65%	87 [75, 99]	91 [76, 106]
Systolic blood pressure	mmHg	0%	118 [103, 134]	115 [99, 133]		21.30%	118 [102, 136]	114 [98, 134]
Temperature	°C	0%	36.89 [36.28, 37.5]	36.67 [36.11, 37.44]		1.07%	36.78 [36.22, 37.33]	36.67 [16, 37.33]
Sodium	mEq/L	0%	139 [136, 142]	139 [136, 142]		25.37%	139 [136, 141]	139 [135.25, 142]
Potassium	mEq/L	0%	4.1 [3.7, 4.4]	4 [3.7, 4.4]		25.36%	4 [3.7, 4.5]	4.1 [3.7, 4.5]
White blood cells	K cells/mcL	0%	12.1 [8.9, 16.3]	12.9 [8.8, 18.2]		0.06%	11.5 [8.4, 15.5]	12.15 [8.5, 17.2]
Respiratory rate	min^− 1^	0%	18 [15, 17]	20 [18, 19]		0.65%	17 [15, 20]	20 [18, 19]
Hematocrit	%	0%	31.4 [28.5, 34.9]	31.6 [28.1, 35.3]		0.04%	32.2 [28.5, 36.5]	31.5 [27.8, 35.5]
Arterial pH		0%	7.38 [7.33, 7.43]	7.37 [7.31, 7.43]		15.23%	7.38 [7.33, 7.43]	7.37 [7.3, 7.43]

ELEC: elective; EMER: emergency; URG: urgent; PaO_2_: arterial partial pressure of oxygen; FiO_2_: fraction of inspired oxygen; GCS: Glasgow Coma Scale; MAP: mean arterial pressure.

### Missing data

Selected predictors were subject to missing values, to a large extent for some of them (up to 25%, see Table 1). Three approaches were compared to handle incomplete data. First, the analysis used the complete-case cohort, by selecting only patients in whom all variables were available for the first time slot and “last observation carried forward” for the following slots. Second, missing values for the 19 selected predictors were imputed using multiple imputation by chained equations with respect to the hierarchical structure of data (time slots within patients) [18, 21], which allowed us to keep additional patients in whom data were available for at least one of these 19 predictors. Third, the set of covariates used to predict ICU mortality was extended to a larger set of clinical and biological variables regardless of preexisting scores, and missing values for all variables were multiply imputed. This third approach considered a large extent of available predictors without limiting the sample size, as would be required by the complete-case analysis. A new set of 70 predictors was defined according to their availability among patients, which allowed us to keep patients for whom data were available for at least one of these 70 predictors. These predictors were selected solely based on their availability, regardless of their expected clinical relevance or collinearity (e.g., several predictors could describe the same measure performed by different devices, Appendix Table A.1). Continuous predictors were log-transformed when required to improve normality. Ten imputed datasets were generated using random-effect linear and logistic regressions for quantitative and binary variables, respectively, and polytomous regression for other categorical variables. Parameters derived from multiple imputation were estimated with their standard errors through the imputed datasets and pooled using Rubin’s rule [22].

### Neural network architectures and statistical analyses

Four neural network architectures were set up to predict mortality in ICU inpatients: a fully connected neural network (FCN), a convolutional neural network (CNN) [23, 24], a bidirectional long short-term memory (LSTM) recurrent neural network [20] and a CNN-LSTM network [17], which concatenated the information from the two previous networks.

A fine-tuning of the hyperparameters was performed for each of the neural networks. Trained on two imputed datasets from the Imputed-19 and the Imputed-70 cohorts, a grid search was performed based on the AUC scores averaged over 5-fold cross-validation to determine the optimal model architecture for both data formats. Different hyperparameter combinations were iteratively tested by varying the number of layers and neurons per layer for the FCN, the number of neurons for the LSTM, and the number of layers and filters, the kernel size and the stride length for the CNN. In addition to these parameters specific to each type of neural network, different learning rates and batch sizes were tested. The Complete-case cohort was analyzed with the same hyperparameters as the Imputed-19 cohort, as the predictors did not differ.

The FCN used 6 hidden layers: the first hidden layer is composed of 150 neurons, with the number of neurons decreasing with each hidden layer. The CNN used three convolutional layers with an increasing number of filters per layer, an average pooling layer, and finally, a fully connected layer allowing the classification done by the model. Its kernel sizes were 3 and 25 for the Imputed-19 and Imputed-70 cohorts respectively with a stride of 1. The LSTM network used a single layer, with 57 and 220 neurons for the Imputed-19 and Imputed-70 cohorts, respectively, with a fully connected output layer. The CNN-LSTM network combined the hyperparameters of the CNN and LSTM networks.

All neural networks used rectified linear unit (ReLU) activation functions in the hidden layers, a dense output layer with two neurons (for two classes) and a sigmoid activation function. The parameters were optimized with a binary cross-entropy loss function, the Adam optimizer [25], a learning rate of 0.001 and a batch size of 128. Observations were weighted according to the outcome group to which they belonged in order to correct the imbalance between these groups [19].

Using the same data, these neural networks were compared with elastic net, a regularized logistic regression approach that combines the penalties of the lasso and ridge methods to control multicollinearity, which commonly occurs in models with large numbers of predictors [26]. The alpha and lambda parameters (relative weight of ridge and lasso penalties and shrinkage parameter, respectively) were optimized using cross-validation over a grid search. The best performances were obtained for an alpha value of 0.001 (i.e., almost considering coefficients from only ridge regression) and a lambda value of 0.5 for all cohorts.

Performances of these predictive models were assessed by evaluating their discrimination and calibration abilities, with the use of multiple 5-fold cross-validation: patients were split into 5 subsets, 4 of which were used for model training and the 5th for performance evaluation. This procedure was repeated so that all 5 subsets were used for evaluation. This 5-fold cross-validation was carried out 10 times with different partitions of the dataset. The models’ ability to discriminate patients at higher risk of death was evaluated using the average area under the ROC curve (AUC). Pairwise AUC comparisons were performed between models using linear mixed models with a random intercept for the cross-validation dataset partition, a method derived from the Hanley and McNeil test to account for the correlation between cross-validation datasets [27]. Fixed effects were tested directly for the complete-case analyses and after pooling with Rubin’s rule for imputed datasets. Calibration was graphically assessed with calibration plots comparing observed and predicted probabilities after rescaling predictions according to the imbalance weights used for model training.

Neural networks were built using Keras version 2.3.1 and the application programming interface of TensorFlow version 2.1.0. All other analyses were performed using R Statistical Software version 4.0.2 (Foundation for Statistical Computing, Vienna, Austria). All tests were two-tailed at the 0.05 significance threshold.

This study followed guidelines from the Transparent Reporting of a multivariable prediction model for Individual Prognosis Or Diagnosis (TRIPOD) statement [28]. The TRIPOD checklist is provided as Appendix Table A.2.

## Results

### Selection of patients

**Fig. 2:**
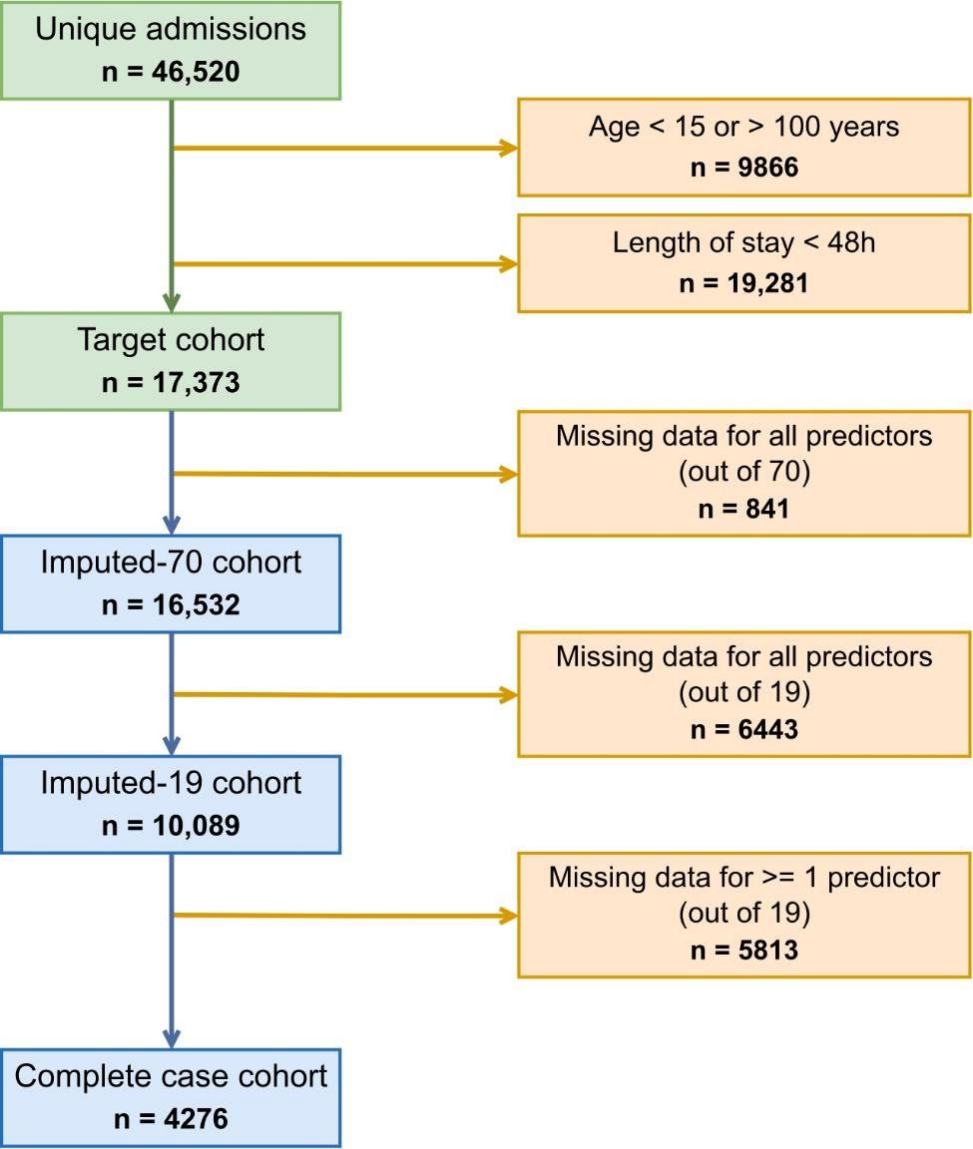
Flowchart for the definition of the three cohorts from the MIMIC-III database. Patients were selected according to age, length of stay ≥ 48 h and available data among the selected predictors

After exclusion of patients aged < 15 or > 100 years, those with missing data on vital status and those staying < 48 h in the ICU, 17,373 patients with unique admission remained in the dataset. According to missing data management, three cohorts were defined. Patients with no missing data in the 19 initially selected predictors, at least for the first time slot, defined the “Complete-case” cohort (n = 4276 patients, 787 deaths). The “Imputed-19” cohort included patients with data available for at least one of the 19 main predictors (n = 10,089 patients, 1499 deaths), whereas the “Imputed-70” cohort did the same with the extended selection of 70 predictors (n = 16,532 patients, 2395 deaths). Models were derived from these two latter cohorts after multiple imputation. Figure 2 summarizes this selection process.

### Models derived from data at admission

Using only admission data from the Complete-case cohort to predict death in patients still in the ICU 48 h after admission, the CNN showed the best performance (AUC = 0.742 ± 0.002, p < 0.001 compared with any other method). The elastic net ranked second (AUC = 0.709 ± 0.002), while the FCN, LSTM and CNN-LSTM all had AUCs under 0.67 (Table 2).

### Time slot duration

Now relying on longitudinal data, still with the Complete-case cohort, neural network performances using time slots of 6- and 12-hour durations were compared. Fully connected networks showed poorer performances than all other models for all time slot durations (p < 0.001 compared with any other method). Models with the best performances were elastic net for 12-hour slots (AUC = 0.789 ± 0.002) and CNN-LSTM for 6-hour slots (AUC = 0.780 ± 0.003), with similar AUCs (p = 0.193). Except for the FCN, which always showed poor performance, all methods using longitudinal data with either 6- or 12-hour slots outperformed the same methods using only admission data (p < 0.001 for all methods).

### Mortality prediction in presence of missing data

Multiple imputation of missing values allowed us to consider a larger set of predictors and to keep larger sample sizes than for complete-case analyses. Table 2 summarizes the predictive performances for all cohorts with 12-hour time slots.

**Table 2:** Performance of elastic net and neural networks to predict ICU mortality (AUC ± SE).

		Complete-case n = 4276			Imputed-19 n = 10,089		Imputed-70 n = 16,532
	Admission data	6-hour slots	12-hour slots		12-hour slots		12-hour slots
Elastic net	0.709 ± 0.002	0.769 ± 0.029	0.785 ± 0.002		0.753 ± 0.024		0.777 ± 0.003
FCN	0.663 ± 0.055	0.521 ± 0.037	0.634 ± 0.049		0.586 ± 0.056		0.542 ± 0.047
CNN	0.742 ± 0.002	0.778 ± 0.005	0.778 ± 0.006		0.751 ± 0.022		0.783 ± 0.003
LSTM	0.602 ± 0.027	0.751 ± 0.028	0.773 ± 0.016		0.764 ± 0.017		0.775 ± 0.019
CNN-LSTM	0.663 ± 0.028	0.780 ± 0.003	0.770 ± 0.009		0.772 ± 0.004		0.779 ± 0.018

Predictions based on admission data or longitudinal data with either 6-hour or 12-hour slots are compared for the Complete-case cohort only. Cohorts defined by missing data management (Complete-case, Imputed-19 or Imputed-70 cohorts) are compared for predictions based on 12-hour slots only.

AUC: area under the ROC curve; SE: standard error; FCN: fully connected network; CNN: convolutional neural network; LSTM: long short-term memory. Imputed-19: missing values imputed for the same 19 predictors as complete-case analyses; Imputed-70: missing values imputed for an additional set of 51 predictors.

**Imputed-19.** Multiple imputation of the 19 previous predictors allowed us to include nearly 2.5 times as many patients as in the complete-case analysis, with similar or slightly weaker performances. The CNN-LSTM (AUC = 0.772 ± 0.004) showed the best performance, close to the complete-case analysis, although no pairwise significant difference in AUC was found between all methods, except the FCN, which showed the weakest performance (p < 0.001 compared with any other method).

**Imputed-70.** Extending the set of predictors to 70 covariates allowed us to include nearly 4 times as many patients as in the complete-case analysis, with similar or slightly better performances except for the FCN. The CNN showed the best performance (AUC = 0.783 ± 0.003), although, as for the Imputed-19 cohort, no significant difference was found among the four best methods.

**Fig. 3:**
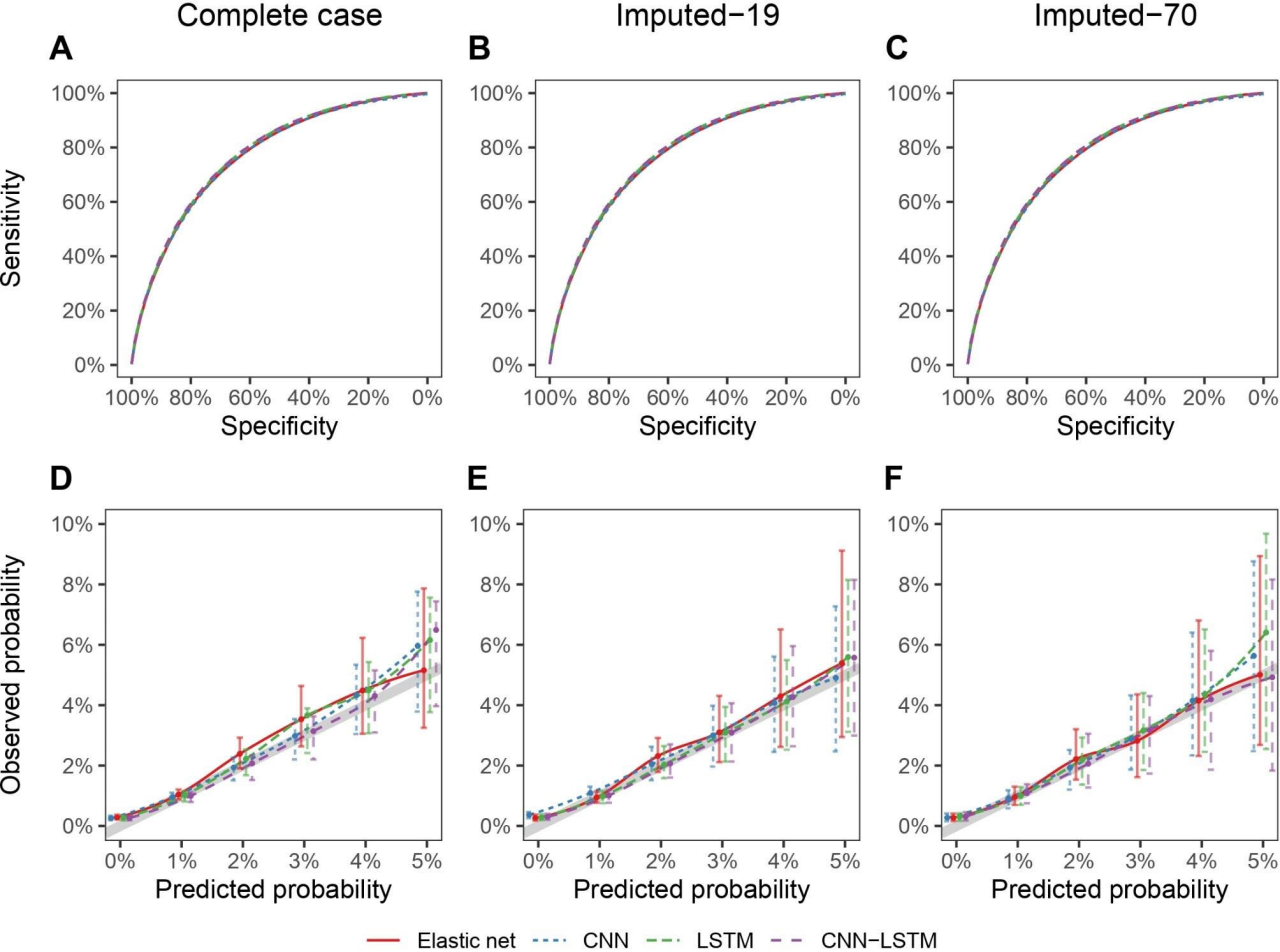
Predictive performances of the elastic net, CNN, LSTM and LSTM-CNN models. Discrimination is represented by the ROC curve (upper figures), and calibration is represented by a smoothed calibration plot showing the observed probabilities (and 95% confidence intervals) according to predicted probabilities (lower figures). The thick gray line shows values expected for a perfect calibration, with observed probabilities equal to predicted probabilities. All estimates are averaged over the 10 repeated 5-fold cross-validation datasets and over the imputed datasets for the Imputed-19 and Imputed-70 cohorts

Figure 3 summarizes the discrimination and calibration performances of the compared modeling methods for all cohorts, except for the FCN, which demonstrated poor performance in all analyses. ROC curves and calibration plots represent the average estimates over the 10 repeated 5-fold cross-validation and over the imputed datasets for the Imputed-19 and Imputed-70 cohorts. All models globally present a fairly satisfactory calibration.

## Discussion

The vast majority of research on risk assessment for ICU mortality concerns short-term stays [6], using only admission data. The increasing availability of electronic health records in health care data warehouses offers new opportunities to closely monitor the evolution of ICU inpatients and to develop prognostic scores relying on a wider range of data, for instance, to detect life-threatening conditions and prevent hospital mortality [29], with promising results regarding specific conditions such as sepsis [30]. This study aimed to assess the relevance of predictive models for mortality in intermediate- or long-term ICU stays, relying on health care data iteratively collected throughout patients’ stays to reevaluate patients’ prognoses. Complementary to usual predictive scores for mortality occurring shortly after admission, such as SAPS II or APACHE II, our analyses focused on patients staying at least 48 h in the ICU.

Here, the mortality risk is continuously updated during patients’ stays, using newly collected data. We nevertheless considered a minimal 12-hour gap between the end of data collection and death, so that the developed models do not identify premortem status but rather leave some time for the medical staff to handle the situation. Considering the massive amount of data possibly available in data warehouses, we focused on statistical approaches likely to integrate a large number of variables, such as deep neural networks and penalized regression models.

Regarding the respective discrimination abilities of models using either 6- or 12-hour time slots, we found that both formats provided similar performances (AUC not significantly different).

Some of the models we developed showed performances very close to the classical predictive scores of ICU mortality [31] or other ICU mortality prediction models based on neural networks [32], yet these latter models use admission data that are not updated throughout patients’ stays. Our first analyses confirmed that the integration of data collected during patients’ stays permitted the identification of patients at higher risk of death better than when relying on baseline data only. Although unsurprising, this result highlights the need to develop and validate predictive scores that could more accurately evaluate patients’ prognoses after some time spent in the ICU.

Missing data are an important issue in clinical studies [33, 34], causing several limitations for complete-case analyses due to the exclusion of patients with missing data: lower sample sizes for the training of models, selection biases if patients without missing data are not representative of the studied population, and the impossibility to apply these models and provide predictions in patients with incomplete data. Using data previously identified as predictive of ICU mortality, our complete-case analysis showed satisfactory results, with an AUC between 0.77 and 0.79 for both penalized regression and convolutional neural networks. However, including patients with data available for all predictors implied selecting a subsample of only 4276 out of the 17,373 in the target cohort (25%), which suggests both a possible selection bias and the inability of our models to infer a mortality risk for patients in whom some of these predictors would be missing.

Multiple imputation by chained equations, using available information for a given patient and the associations between variables derived from the whole sample [35], appeared as a promising option to address these issues. A first attempt to impute data for these 19 predictors (Imputed-19 cohort) allowed us to include a larger sample size (10,089 patients, 58% of the target cohort) without degrading predictive performances. More interestingly, data imputation considering a larger set of potential predictors (Imputed-70 cohort) allowed us to include an even larger sample size (16,532 patients, 95% of the target cohort) with slightly better performances than for the Complete-case cohort. The excluded patients were those for whom no data were available at admission, and it is therefore difficult to determine how they differed from the included patients. For similar reasons, data imputation relies on a hypothesis of “missing completely at random” or “missing at random” mechanisms, and we cannot rule out a “missing not at random” mechanism (the probability of missing values depends on unobserved characteristics). In such a context, our models would yield biased estimates in patients with data missing for specific predictors. However, our cross-validation procedure used to estimate model performance captures the inaccuracy that could result from the missing data pattern, and reported results already integrate this possible source of error. This robustness to missing data imputation is insightful, as it suggests that predictive models might be developed in ICUs admitting patients with specific conditions and provide prognosis predictions for all patients with a higher precision as available information accumulates.

Although deep neural networks are increasingly popular for handling massive data [16, 36], they did not outperform more conventional penalized regressions in our study. An explanation might be that available data did not take full advantage of the time slot format [37]: although some predictors, such as vital signs or blood tests, were frequently updated, medical conditions likely to dramatically impact prognosis, such as the occurrence of shock or organ failure, were collected retrospectively but not on time to be used as a predictor. This limitation is due to the nature of the MIMIC-III database and may be present in other health care data warehouses, yet we assume that a timely collection of medical diagnoses and relevant symptoms may be insightful to enhance predictive performances.

Our study has several strengths, including a novel approach to integrate updated information on patients’ characteristics to estimate their prognosis more accurately and the additional opportunity to use this information even when data are partially missing. The 12-hour gap between the collection of predictors and the occurrence of the predicted event also appears to be clinically relevant, as it allows the medical staff to take preventive measures whenever possible. Depending on the specificities of each ICU, similar predictive models could be developed for outcomes other than mortality, e.g., the occurrence of shock, organ failure or multiple organ dysfunction.

Several limitations must also be noted. First, this study must be seen as a “proof of concept” for a novel predictive modeling framework, but we do not expect that inferring our models’ parameters to other ICUs with specific patients and data collections might yield meaningful predictions. We nevertheless assume that using the same modeling approaches in a new setting may produce models with similar performances. Additionally, contrary to exponentiated regression coefficients of elastic net models that can be directly interpreted as odds ratios for the considered predictors, the “black box” nature of neural networks does not allow easy identification of specific predictors associated with a higher risk of mortality. These models must therefore be seen as global “alert systems” rather than as a tool likely to identify specific complications. Other statistical learning approaches, such as tree ensemble models, may also show satisfactory performances and should be evaluated in further studies. Finally, a technical limitation lies in the possibility of data collection and automated analysis of health care data almost in real time. Although very few ICUs might present this ability, the current development of health care data warehouses worldwide may enhance feasibility.

## Conclusion

This proof-of-concept study supports that automated analysis of electronic health records can be of great interest throughout patients’ stays as a surveillance tool likely to provide early detection of life-threatening conditions. Such predictive models may be insightful as a continuation of usual mortality predictive scores relying solely on admission data, especially regarding long hospital stays. Although this framework relies on a large set of predictors, it is robust to data imputation and may be effective early after admission, when data are still scarce. Further studies would be needed to evaluate the applicability and interest of this approach in ICUs addressing specific populations or medical conditions.

### Electronic supplementary material

Below is the link to the electronic supplementary material.

Supplementary Material 1

## Data Availability

The datasets analyzed during the current study are available in the MIMIC-III repository, https://mimic.physionet.org/. Researchers must complete a suitable training program in human research subject protections and HIPAA regulations before they can apply for permission to access it.

## List of abbreviations

APACHE II: Acute Physiology And Chronic Health Evaluation II
AUC: Area under the ROC curve
CNN: Convolutional neural network
FCN: Fully connected neural network
ICU: Intensive care unit
LSTM: Long short-term memory
MIMIC-III: Medical Information Mart for Intensive Care III
ReLU: Rectified linear unit
SAPS II: Simplified Acute Physiology Score II
SOFA: Sequential Organ Failure Assessment Score
TRIPOD: Transparent Reporting of a multivariable prediction model for Individual Prognosis Or Diagnosis
