# Supplementary material for: Updating mortality risk estimation in intensive care units from high-dimensional electronic health records with incomplete data
Source: BMC Med Inform Decis Mak. 2023 Aug 30;23:170. doi: 10.1186/s12911-023-02264-7 (PMC10466694; doi:10.1186/s12911-023-02264-7)
Supplement: Supplementary file 1 — Supplementary Material 1 [file 12911_2023_2264_MOESM1_ESM.docx]

|  |  | | Missing data | Survivorsn = 14,137 | Non-survivorsn = 2,395 |
| --- | --- | --- | --- | --- | --- |
| Male gender | |  | 55.7% | 57.1% | 53.8% |
| Admission type | |  | 0% | ELEC: 2063 (14.6%)EMER: 11624 (82.2%)URG: 450 (3.2%) | ELEC: 111 (4.6%)EMER: 2196 (91.7%)URG: 88 (3.7%) |
| Previous ward | |  | 0% | 17 classes | 17 classes |
| Current ward | |  | 0% | 18 classes | 18 classes |
| Marital status | |  | 60.5% | single: 1600 (28.3%)married: 2948 (52.1%)divorced: 409(7.2%)widowed: 695 (12.3%)life partner: 2 (0.04%) | single: 235 (26.8%)married: 443 (52.1%)divorced: 63 (7.2%)widowed: 135 (15.4%)life partner: 0 (0%) |
| History of falling within 3 months | |  | 60.9% | 1394 (25.1%) | 303 (33.3%) |
| Secondary diagnosis | |  | 60.9% | 5049 (90.9%) | 852 (93.7%) |
| Ambulatory aid | |  | 60.9% | bed rest: 5492 (98.9%)walker: 51 (0.9%)furniture: 10 (0.2%) | bed rest: 908 (99.9%)walker: 1 (0.1%)furniture: 0 (0%) |
| IV saline lock | |  | 60.9% | 5324 (95.9%) | 880 (96.8%) |
| Gait transferring | |  | 60.9% | bed rest: 4141 (74.5%)weak: 1176 (21.2%)impaired: 237(4.3%) | bed rest: 881 (96.9%)weak: 12(1.3%)impaired: 16 (1.8%) |
| Mental status | |  | 60.9% | oriented to own ability: 3355 (60.4%)forgets limitations: 2198 (39.6%) | oriented to own ability: 101(11.1%)forgets limitations: 808 (88.9%) |
| Age | | years | 0% | 64.86 [52.18, 76.49] | 70.93 [58.42, 80.25] |
| GCS total | | points | 38.92% | 11 [6, 15] | 10 [6, 15] |
| GCS eye opening 1 | | points | 38.92% | 3 [1, 4] | 3 [1, 4] |
| GCS eye opening 2 | | points | 60.89% | 4 [1, 4] | 3 [1, 4] |
| GCS verbal response 1 | | points | 60.89% | 4 [1, 5] | 1 [1, 5] |
| GCS verbal response 2 | | points | 38.92% | 1 [1, 5] | 1 [1, 5] |
| GCS motor response 1 | | points | 60.89% | 6 [4, 6] | 6 [4, 6] |
| GCS motor response 2 | | points | 38.92% | 6 [4, 6] | 5 [4, 6] |
| Pain | | level | 61.61% | 0 [0, 3] | 0 [0, 0] |
| Braden score | | points | 39.00% | 14 [12, 16] | 13 [12, 15] |
| Braden sensory perception | | points | 60.92% | 3 [2, 4] | 3 [2, 3] |
| Braden moisture | | points | 60.92% | 4 [3, 4] | 4 [3, 4] |
| Braden activity | | points | 60.92% | 1 [1, 1] | 1 [1, 1] |
| Braden mobility | | points | 60.92% | 3 [2, 3] | 2 [2, 3] |
| Braden nutrition | | points | 60.92% | 2 [2, 3] | 2 [2, 2] |
| Braden friction shear | | points | 60.92% | 2 [2, 3] | 2 [2, 2] |
| Systolic BP 1 | | mmHg | 61.47% | 113 [101, 122] | 109 [96, 120] |
| Systolic BP 2 | | mmHg | 52.05% | 119 [104, 136] | 116 [99, 135] |
| Systolic BP 3 | | mmHg | 40.04% | 116 [101, 133] | 112 [98, 130] |
| Diastolic BP 1 | | mmHg | 60.99% | 63 [54, 75] | 62 [51, 74] |
| Diastolic BP 2 | | mmHg | 52.05% | 61 [53, 70] | 59 [50, 69] |
| Diastolic BP 3 | | mmHg | 40.05% | 57 [48, 68] | 55 [46, 66] |
| Mean BP 1 | | mmHg | 60.98% | 76 [66, 88] | 73 [63, 87] |
| Mean BP 2 | | mmHg | 40.06% | 76 [66, 87] | 73.67 [63, 85] |
| Mean BP 3 | | mmHg | 52.01% | 80 [71, 92] | 78 [68, 91] |
| Glucose 1 | | mg/dL | 38.22% | 135 [110, 170] | 141 [112, 182] |
| Glucose 2 | | mg/dL | 55.75% | 129 [107, 158] | 135 [107, 174] |
| Glucose 3 | | mg/dL | 48.09% | 135 [112, 170] | 145 [115, 186] |
| Heart rate 1 | | min^-1^ | 38.92% | 87 [75, 99] | 91 [76, 106] |
| Heart rate 2 | | min^-1^ | 60.80% | 86 [74, 100] | 89 [76.75, 105] |
| Respiratory rate 1 | | min^-1^ | 60.82% | 18 [15, 22] | 20 [16, 25] |
| Respiratory rate 2 | | min^-1^ | 38.92% | 17 [14, 21] | 20 [16, 24] |
| Potassium 1 | | mEq/L | 38.22% | 4 [3.7, 4.5] | 4.1 [3.7, 4.6] |
| Potassium 2 | | mEq/L | 55.75% | 4.1 [3.7, 4.5] | 4.1 [3.7, 4.6] |
| HCO_3_^-^ | | mEq/L | 0.09% | 23 [21, 26] | 22 [19, 26] |
| FiO_2_ | | proportion | 52.26% | 0.6 [0.5, 1] | 0.7 [0.5, 1] |
| O_2_ saturation 1 | | % | 60.82% | 98 [96, 100] | 98 [95, 100] |
| O_2_ saturation 2 | | % | 38.92% | 99 [97, 100] | 98 [96, 100] |
| O_2_ flow | | L/min | 44.56% | 5 [3, 12] | 5 [3, 12] |
| Arterial base excess | | mEq/L | 68.89% | 4 [3, 5] | 4 [3, 5] |
| Arterial CO_2_ | | mEq/L | 48.17% | 25 [22, 28] | 23 [19, 28] |
| Arterial PaCO_2_ | | mmHg | 48.17% | 40 [35, 46] | 38 [33, 46] |
| Arterial PaO_2_ | | mmHg | 48.16% | 163 [98, 285] | 128 [83, 209] |
| Red blood cells | | 10^6^ cells/mm^3^ | 38.31% | 3.57 [3.15, 4.05] | 3.48 [3.04, 3.95] |
| White blood cells | | 10^3^ cells/mm^3^ | 0.27% | 11.2 [8.1, 15.1] | 12.0 [8.3, 17.0] |
| Hemoglobin | | g/dL | 0.07% | 10.8 [9.5, 12.3] | 10.6 [9.3, 12] |
| Hematocrit | | % | 0.05% | 32.2 [28.4, 36.5] | 31.6 [27.7, 35.9] |
| Platelets | | 10^3^ cells/mm^3^ | 0.07% | 199 [146, 269] | 191 [117, 175] |
| BUN | | mg/dL | 0.04% | 18.2 [12.9, 28.2] | 25.7 [17.0, 43.6] |
| Calcium | | mg/dL | 2.03% | 8.3 [7.7, 8.8] | 8.2 [7.6, 8.7] |
| Chloride | | mEq/L | 0.06% | 106 [102, 109.25] | 105 [100, 109] |
| Blood creatinine | | mg/dL | 0.13% | 0.89 [0.71, 1.29] | 1.10 [0.79, 1.91] |
| Magnesium | | mg/dL | 0.19% | 1.9 [1.7, 2.2] | 1.9 [1.7, 2.2] |
| Sodium | | mEq/L | 0.05% | 139 [136, 141] | 139 [135, 142] |
| Phosphorous | | mg/dL | 1.86% | 3.47 [2.82, 4.17] | 3.72 [2.88, 4.68] |
| Arterial pH | |  | 47.99% | 7.38 [7.33, 7.43] | 7.37 [7.3, 7.43] |
| Temperature | | °C | 0.47% | 36.78 [36.22, 37.33] | 36.61 [36, 37.22] |
| Weight | | kg | 4.48% | 80 [67.2, 94.6] | 75.5 [63, 90.4] |
| Previous weight | | kg | 49.48% | 82.1 [69, 97.5] | 78 [65.2, 93.9] |

**Appendix table A.1 Characteristics of patients in the Imputed-70 cohort at admission (median [Q1, Q3]).**

PaO_2_: Arterial partial pressure of oxygen; FiO_2_: Fraction of inspired oxygen; GCS: Glasgow Coma Scale; BP: Blood pressure; PaO_2_: Partial pressure of oxygen; PaCO_2_: Partial pressure of carbon dioxide; BUN: Blood Urea Nitrogen


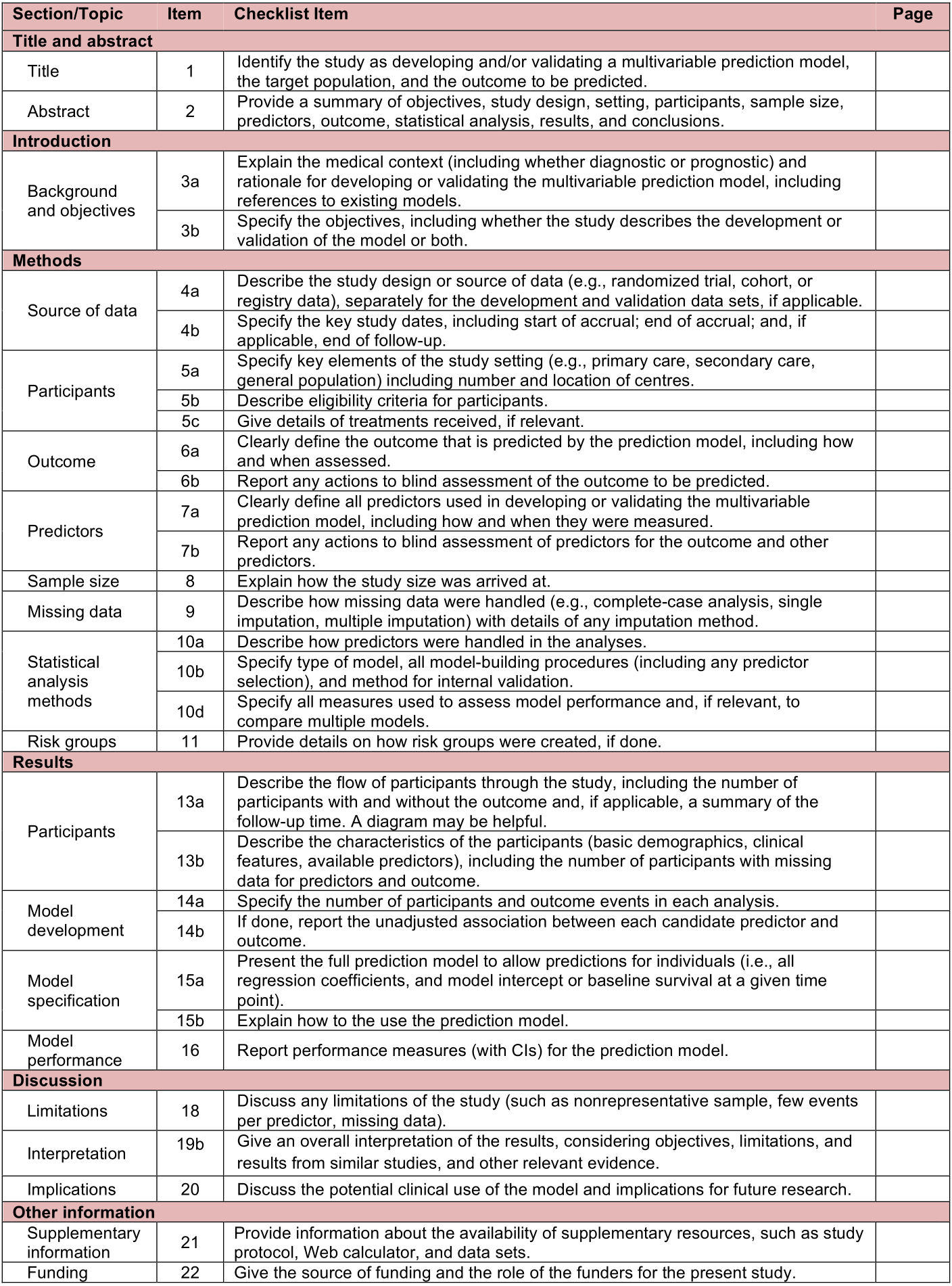


19

19

15 - 17

18

12 - 14 14

-

11

10 - 11

5 - 6

11

8

5

18

7

10

9 - 10

5 - 7

-

6

5

-

-

-

-

-

-

5

4

3-4

1-2

1

**Appendix table A.2 TRIPOD checklist of items of interest in study developing a multivariable prediction model for prognosis**
